# Items

**Published:** 1907-03

**Authors:** 


### ITEMS.

A board of officers will be convened to meet at the Bureau of Public Health and Marine Hospital Service, 3 B street SE., Washington, D. C., Monday, April 15, 1907, at 10 o'clock a. m., for the purpose of examining candidates for admission to the grade of assistant surgeon in the Public Health and Marine-Hospital Service. For further information, or for invitation to appear before the board of examiners, address Surgeon-General. Public Health and Marine-Hospital Service, Washington, D. C.

Preliminary examinations for appointment of Assistant Surgeons in the Army will be held on April 29 and July 29, 1907, at points to be hereafter designated. Permission to appear for examination can be obtained upon application to the Surgeon General, U. S. Army, Washington, D. C., from whom full information concerning the examination can be procured.

